# Quantitative Analysis and Biological Efficacies regarding the Neuroprotective and Antineuroinflammatory Actions of the Herbal Formula Jodeungsan in HT22 Hippocampal Cells and BV-2 Microglia

**DOI:** 10.1155/2017/6360836

**Published:** 2017-12-17

**Authors:** Yu Jin Kim, Hye-Sun Lim, Bu-Yeo Kim, Chang-Seob Seo, Soo-Jin Jeong

**Affiliations:** ^1^Herbal Medicine Research Division, Korea Institute of Oriental Medicine, Daejeon 34054, Republic of Korea; ^2^College of Pharmacy, Chungnam National University, Daejeon 34134, Republic of Korea; ^3^K-Herb Research Center, Korea Institute of Oriental Medicine, Daejeon 34054, Republic of Korea; ^4^Korean Medicine Life Science, University of Science & Technology, Daejeon 34113, Republic of Korea

## Abstract

Jodeungsan (JDS) is a traditional herbal formula that comprises seven medicinal herbs and is broadly utilized to treat hypertension, dementia, and headache. However, the effects of JDS and its herbal components on neurodegenerative diseases have not been reported. We examined the inhibitory effects of JDS and its seven components on neuronal cell death and inflammation using HT22 hippocampal cells and BV-2 microglia, respectively. Among its seven herbal components,* Uncaria sinensis* (US),* Chrysanthemum morifolium* (CM),* Zingiber officinale* (ZO),* Pinellia ternata* (PT),* Citrus unshiu* (CU), and* Poria cocos* (PC) exhibited significant neuroprotective effects in HT22 cells. In BV-2 cells, JDS significantly suppressed the production of tumor necrosis factor-alpha (TNF-*α*) and interleukin-6 (IL-6), indicating the antineuroinflammatory activity of JDS. In addition, the herbal extracts from ZO,* Panax ginseng* (PG), PT, CU, and PC exhibited inhibitory effects on the inflammatory response in microglia. These data imply that the JDS effect on neurodegeneration occurs via coordination among its seven components. To establish a quality control for JDS, a simultaneous analysis using five standard compounds identified hesperidin (37.892 ± 1.228 mg/g) as the most abundant phytochemical of JDS. Further investigation of the combinatorial activities of two or more standard compounds will be necessary to verify their antineurodegenerative regulatory mechanisms.

## 1. Introduction

Neurodegenerative disease is a medical condition that is characterized by the progressive loss of neural tissues. Parkinson's, Alzheimer's, and Huntington's diseases and their related disorders are neurodegenerative diseases [1, 2]. Aging is one of the strongest risk factors for neurodegenerative diseases [3]. Senior populations are increasing in modern society, which leads to the increase in the prevalence of neurodegenerative diseases. Thus, these diseases are considered as significant challenges for both preclinical and clinical investigations. However, although abundant studies have tried to develop antineurodegeneration drugs, there are no powerful and effective agents; therefore, these diseases are currently incurable. Because the pathogenesis of neurodegenerative diseases is complex and neuronal and nonneuronal cells in neurovascular cell units coordinately participate in disease progression [4, 5], a single molecule that targets these conditions is not a suitable approach in new drug development for neurodegenerative diseases. Recently, herbal medicines have been regarded as appropriate materials based on their positive aspect of having multiple components and multiple targets [6, 7]. Several articles have suggested the possibility that herbal medicines are useful therapeutic agents for neurodegenerative diseases [8–10].

Jodeungsan (JDS), also called Chotosan in Japan and Diaoteng San in China, is a traditional herbal formula that comprises seven medicinal herbs,* Uncaria sinensis* (US),* Chrysanthemum morifolium* (CM),* Zingiber officinale* (ZO),* Panax ginseng* (PG),* Pinellia ternata* (PT),* Citrus unshiu* (CU), and* Poria cocos* (PC). Clinically, JDS has been prescribed for age-related diseases such as hypertension [11], dementia and cognitive impairment [12, 13], and chronic headache [14]. Several clinical reports demonstrated the potential of JDS for treating dementia, one of the most common neurodegenerative diseases. Suzuki et al. reported that a double-blinded, randomized, and placebo-controlled study addressed the positive aspect of JDS on cognitive function and activities of daily living in patients with dementia [12]. Yamaguchi et al. reported that JDS improved the event-related brain potential in stroke patients with cognitive impairment [15]. Pharmacobiological evidence of the action of JDS against dementia has been described in* in vitro* or* in vivo* models [16–18]. However, most articles have described the anti-dementia effect of JDS itself, but not that of each of its herbal components.

Our study aimed to investigate the effect of JDS and its seven herbal components on neurodegenerative events using hippocampal and microglia cell lines. We also performed a simultaneous analysis of five standard compounds to improve the quality control of JDS.

## 2. Materials and Methods

### 2.1. Plant Materials

The seven crude herbal medicines that form JDS, Uncariae Ramulus et Uncus, Chrysanthemi Flos, Zingiberis Rhizoma, Ginseng Radix Alba, Pinelliae Tuber, Citri Unshii Pericarpium, and Poria Sclerotium, were purchased from the Kwangmyungdang herbal market (Ulsan, South Korea). A voucher specimen has been deposited at the Herbal Medicine Research Division, Korea Institute of Oriental Medicine.

### 2.2. Chemicals and Reagents

**Figure 1:**
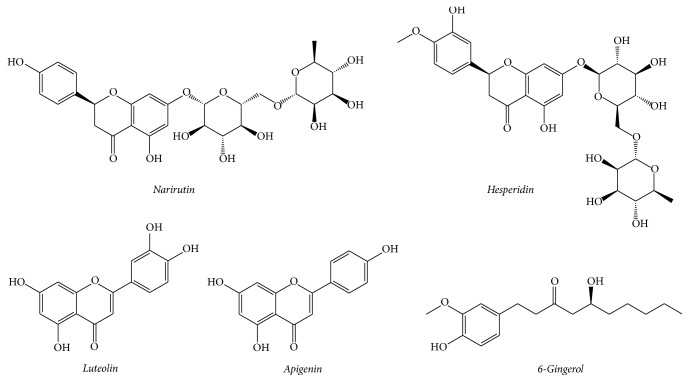
Chemical structures of the five standard compounds of JDS.

The standard components, luteolin and apigenin, were purchased from ChemFaces Biochemical Co., Ltd. (Wuhan, China), and hesperidin, 6-gingerol, and narirutin were purchased from Biopurify Phytochemicals (Chengdu, China). The chemical structures of the standard components are shown in Figure 1. The purity of these standard components was ≥98.0%, as assessed using high-performance liquid chromatography (HPLC) analysis. The HPLC-grade solvents, acetonitrile and water, were purchased from J. T. Baker Chemical Co. (Phillipsburg, NJ, USA), and the analytical-grade reagent trifluoroacetic acid (TFA) was purchased from Sigma-Aldrich (St. Louis, MO, USA).

### 2.3. Apparatus and Chromatographic Conditions

The quantitative analysis was conducted using a Waters Alliance e2695 system (Waters Corp, Milford, MA, USA) equipped with a pump, degasser, column oven, autosampler, and photodiode array detector (#2998; Waters Corp). The data were acquired and processed using Empower software (version 3; Waters Corp). The chromatographic separation of the seven bioactive components was carried out at room temperature using Luna C_18_ analytical columns (250 × 4.6 mm, 5 *μ*m), supplied by Phenomenex (Torrance, CA, USA), with a gradient solvent system of 0.1% (v/v) aqueous TFA (A) and acetonitrile (B). The elution conditions were as follows: 15%–40% B for 0–30 min, 40%–100% B for 30–40 min, and 100% B for 40–47 min. The flow rate was 1.0 mL/min and the injection volume was 10 *μ*L. The ultraviolet (UV) wavelength for detecting components was 236 nm for narirutin, hesperidin, and 6-gingerol and 263 nm for luteolin and apigenin.

### 2.4. Preparation of Standard Solutions

The five reference standards were weighed accurately, dissolved in methanol at 1.0 mg/mL, and stored at <4°C. The stock solutions were diluted to yield a series of standard solutions with different concentrations for quantitative analysis.

### 2.5. Preparation of Sample Solutions

The seven dried crude herbal medicines, Uncariae Ramulus et Uncus, Chrysanthemi Flos, Zingiberis Rhizoma, Ginseng Radix Alba, Pinelliae Tuber, Citri Unshii Pericarpium, and Poria Sclerotium, were mixed as indicated in Table 1 (51 g) and extracted twice with 70% ethanol (306 mL) by refluxing for 2 h. The extracted solution was filtered through a filter paper (5 *μ*m) and evaporated using a rotary evaporator (EYELA N-1000, Rikakikai Co., Tokyo, Japan) under vacuum to dryness (8.164 g). The yield of JDS extract was 16%. For simultaneous determination of the powdered JDS extract, the 70% ethanol extract of the JDS was weighed accurately and dissolved in methanol at 20 mg/mL. The sample solution was filtered through a syringe filter (0.45 *μ*m) for HPLC analysis.

### 2.6. Calibration Curve and Determination of the Limits of Detection and Quantification

The calibration curves of all components were calculated from the peak areas of the standard solutions at different concentrations. The tested concentration ranges were as follows: narirutin (12.5–400 *μ*g/mL), hesperidin (31.25–500 *μ*g/mL), luteolin (1.5625–25 *μ*g/mL), apigenin (0.78125–25 *μ*g/mL), and 6-gingerol (3.125–50 *μ*g/mL). These solutions were measured in triplicate for the calibration curves. The limit of detection (LOD) and limit of quantification (LOQ) for the five standard components were calculated, using the slope of the calibration curve and the standard deviation (SD) of the intercept, as follows: LOD = 3.3 × (SD of the response/slope of the calibration curve); and LOQ = 10 × (SD of the response/slope of the calibration curve).

### 2.7. Herbal Extract Treatment and Cytotoxicity Assay

BV-2 and HT22 cells were maintained in Dulbecco's modified Eagle's medium (Hyclone/Thermo, Rockford, IL, USA), supplemented with 10% fetal bovine serum (Hyclone/Thermo) and penicillin/streptomycin in a 5% CO_2_ at 37°C. HT22 cells were cotreated with the herbal extract and hydrogen peroxide (H_2_O_2_, 250 *μ*M; Sigma-Aldrich) for 6 h. BV-2 cells were pretreated with various concentrations of each herbal extract for 2 h and treated with lipopolysaccharide (LPS, 1 *μ*g/mL; Sigma-Aldrich) for additional 22 h.

The cytotoxicity test was performed as described previously [19]. In brief, BV-2 and HT22 cells were plated on 96-well microplates at a density of 3 × 10^4^/well and 5 × 10^3^/well, respectively. Cells were treated with various concentrations of each herbal extract for 24 h. Cell counting kit-8 (CCK-8) solution (Dojindo, Kumamoto, Japan) was added, and the cells were incubated for 4 h. The absorbance was read at 450 nm on an Epoch Microplate Spectrophotometer (BioTek Instruments, Inc., Winooski, VT, USA). The cell viability was calculated using the following equation:

(1) Cell  viability%=Mean  OD  in  drug−treated  cellsMean  OD  in  untreated  cells×100.

### 2.8. Measurement of Cytokine Production

Culture supernatants were collected from the cells treated with various concentrations of each herbal extract in the presence of LPS. An enzyme-linked immunosorbent assay (ELISA) kit from R&D systems (Minneapolis, MN, USA) was used in accordance with the manufacturer's instructions. The concentration of each sample was calculated according to the standards provided with the kits.

### 2.9. Statistical Analysis

The data are expressed as the mean ± SEM. Data were analyzed using one-way analysis of variance and Dunnett's multiple comparisons test. *P* < 0.05 was considered significant.

## 3. Results

### 3.1. Optimization of HPLC Separation

**Figure 2:**
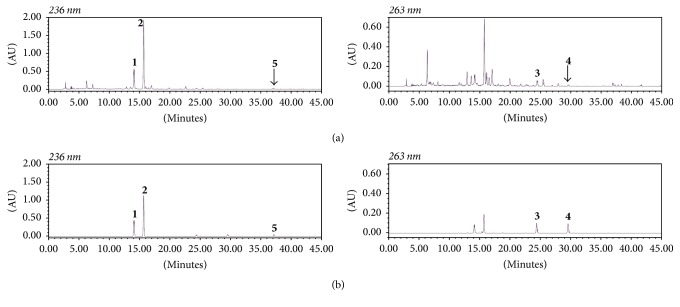
HPLC chromatograms of the 70% ethanol extract of JDS (a) and a standard mixture (b) at 236 nm and 263 nm. Narirutin** (1)**, hesperidin** (2)**, luteolin** (3)**, apigenin** (4)**, and 6-gingerol** (5)**.

An HPLC analytical method was established for the simultaneous separation of the five standard components from the 70% ethanol extract of JDS. As a result, a completely separated chromatogram was obtained within 40 min using two mobile phases consisting of 0.1% (v/v) aqueous TFA (a) and acetonitrile (b). The UV wavelength used for quantitative analysis was 236 nm for narirutin, hesperidin, and 6-gingerol and 263 nm for luteolin and apigenin. Under these established HPLC methods, the retention times of narirutin, hesperidin, luteolin, apigenin, and 6-gingerol were 14.09, 15.67, 24.37, 29.52, and 37.09 min, respectively. HPLC chromatograms of the 70% ethanol extract of JDS and the standard mixture are shown in Figure 2.

### 3.2. Linearity, LOD, and LOQ

The linear relationships between the peak area (*y*) and concentration (*x*, *μ*g/mL) of each component were expressed by the regression equations (*y* = *ax* + *b*), as shown in Table 2. The calibration curves for the five components showed good linearity (*r*^2^ ≥ 0.9998). The LOD and LOQ for the tested components were 0.013–1.054 *μ*g/mL and 0.041–3.194 *μ*g/mL, respectively.

### 3.3. Determination of the Five Standard Components of the JDS Extract

The HPLC analytical method established here was applied to the simultaneous quantification of the five components of the extracted JDS sample. The relative amount of the five standard components ranged from 0.061 to 37.892 mg/g and the results of the analysis are summarized in Table 3. Among the five components of JDS, hesperidin was the most abundant.

### 3.4. Cytotoxicity of JDS and Its Seven Herbal Components against HT22 and BV-2 Cells

To determine the cytotoxic effects of JDS and its seven components, a CCK assay was used. HT22 or BV-2 cells were treated with various concentrations of each herbal extract for 24 h and the cell viability was measured. The results are shown in Table 4. Cell treatment with each extract was performed in the nontoxic concentration range in all subsequent experiments.

### 3.5. Neuroprotective Effect of JDS and Its Seven Herbal Components in H_2_O_2_-Damaged HT22 Hippocampal Cells

**Figure 3:**
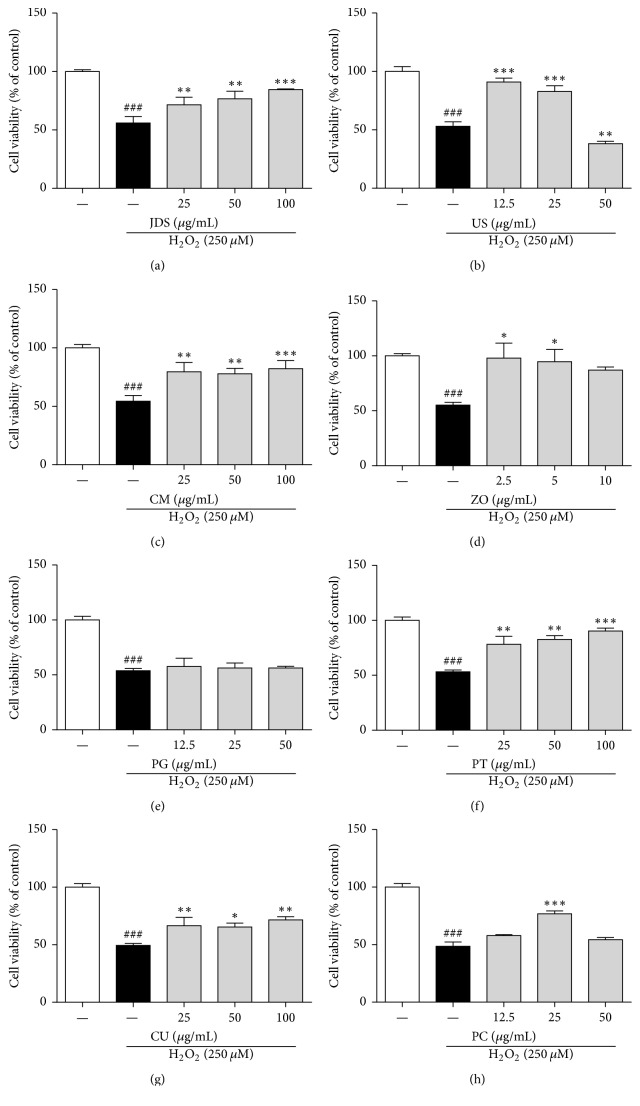
Neuroprotective effect of JDS and its herbal components in H_2_O_2_-treated HT22 cells. Cells were seeded on 96-well plates and cotreated with various concentrations of JDS and its seven components, and H_2_O_2_ (250 *μ*M) for 6 h. Cell viability was assessed using the CCK-8 assay. Results are shown for JDS (a), US (b), CM (c), ZO (d), PG (e), PT (f), CU (g), and PC (h). The results are expressed as the mean ± SEM of three independent experiments. ^###^*P* < 0.001 versus vehicle control cells, ^*∗*^*P* < 0.05, ^*∗∗*^*P* < 0.01, and ^*∗∗∗*^*P* < 0.001 versus H_2_O_2_-treated cells.

To examine the protective effects of JDS and its seven components in neuronal cells, the HT22 hippocampal cell line was used. Neuronal cell damage was induced by exposing cells to H_2_O_2_, followed by treatment with various concentrations of the herbal extracts. Treatment with H_2_O_2_ alone significantly reduced cell viability in comparison with the untreated control. The JDS extract significantly inhibited the H_2_O_2_-mediated cell damage (Figure 3(a)). Among the seven herbal components, US (b), CM (c), ZO (d), PT (f), CU (g), and PC (h) showed protective activity against H_2_O_2_-mediated cell damage.

### 3.6. Inhibitory Effect of JDS and Its Seven Herbal Components on Proinflammatory Cytokine Production in LPS-Stimulated BV-2 Microglia

**Figure 4:**
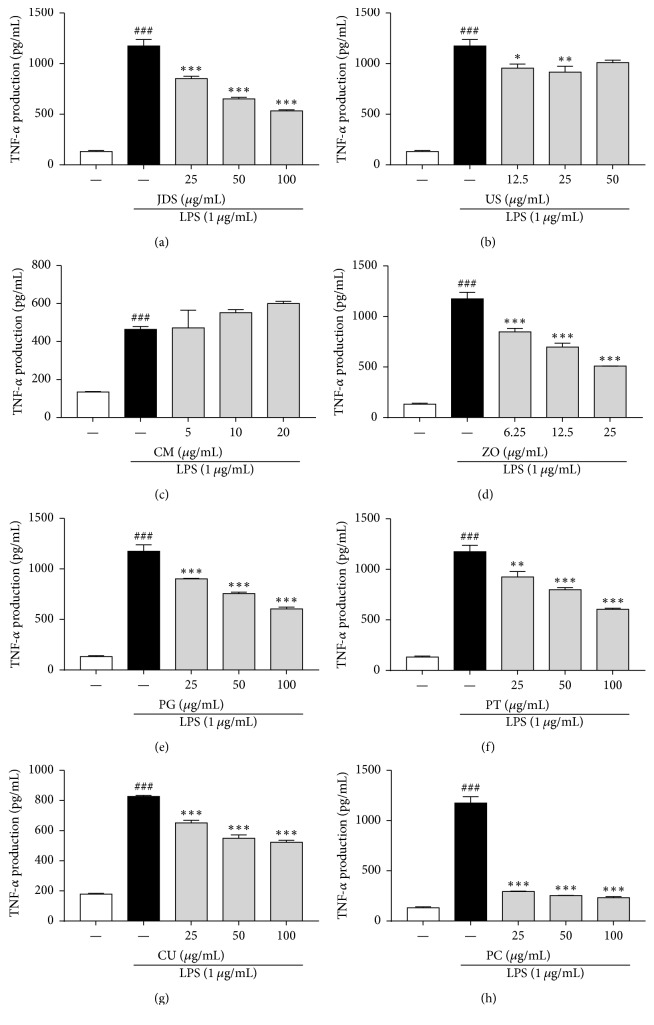
Effects of JDS and its herbal components on TNF-*α* production in LPS-stimulated BV-2 cells. Cells were pretreated with various concentrations of JDS and its seven components for 2 h and then stimulated with LPS (1 *μ*g/mL) for an additional 22 h. TNF-*α* production was determined using an ELISA kit. Results are shown for JDS (a), US (b), CM (c), ZO (d), PG (e), PT (f), CU (g), and PC (h). The results are expressed as the mean ± SEM of three independent experiments. ^###^*P* < 0.001 versus vehicle control cells, ^*∗*^*P* < 0.05, ^*∗∗*^*P* < 0.01, and ^*∗∗∗*^*P* < 0.001 versus LPS-treated cells.

**Figure 5:**
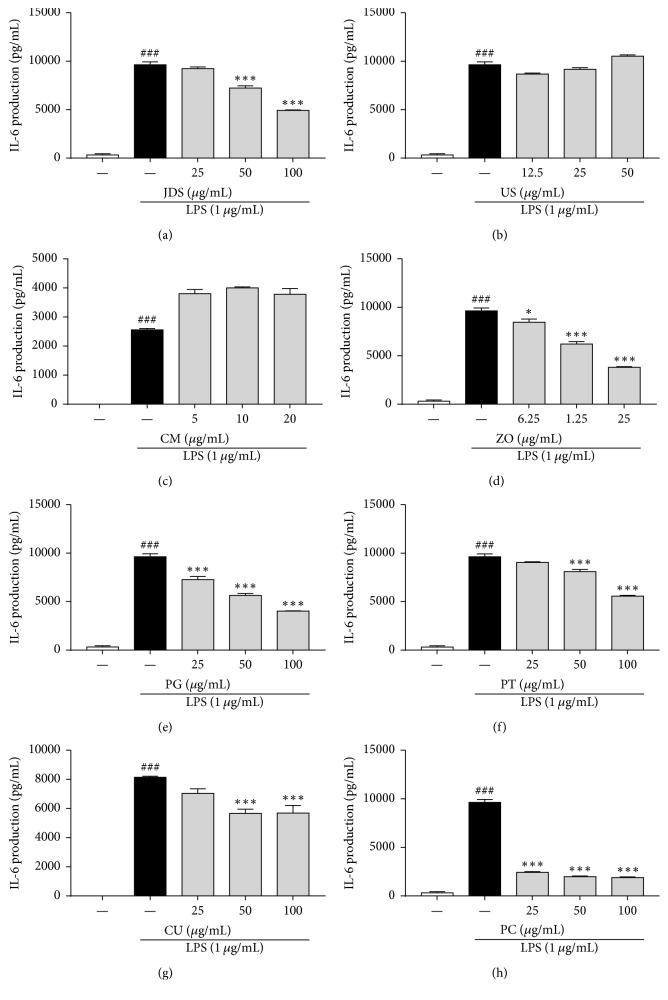
Effects of JDS and its herbal components on IL-6 production in LPS-stimulated BV-2 cells. Cells were pretreated with various concentrations of JDS and its seven components for 2 h and then stimulated with LPS (1 *μ*g/mL) for an additional 22 h. IL-6 production was determined using an ELISA kit. Results are shown for JDS (a), US (b), CM (c), ZO (d), PG (e), PT (f), CU (g), and PC (h). The results are expressed as the mean ± SEM of three independent experiments. ^###^*P* < 0.001 versus vehicle control cells, ^*∗*^*P* < 0.05 and ^*∗∗∗*^*P* < 0.001 versus LPS-treated cells.

Next, we investigated whether JDS and its seven herbal components suppress the production of proinflammatory cytokines, such as tumor necrosis factor-alpha (TNF-*α*) and interleukin-6 (IL-6), in LPS-stimulated BV-2 cells. As shown in Figures 4 and 5, TNF-*α* and IL-6 levels increased in the culture media of LPS-stimulated BV-2 cells, and these increases were significantly reduced by the treatment with JDS (Figures 4(a) and 5(a)) and its components ZO (d), PG (e), PT (f), CU (g), and PC (h). PC had the most significant inhibitory effect on the LPS-stimulated TNF-*α* and IL-6 production (Figures 4(h) and 5(h)).

## 4. Discussion

Neuronal cell death plays a crucial role in neurodegeneration [20]. In general, the induction of neuronal cell death in neurodegenerative diseases, including Alzheimer's, Parkinson's, and Huntington's diseases, is controlled by pathways related to mitochondrial dysfunction and oxidative stress [21, 22]. Neuronal cell death is associated with neuroinflammation in the central nervous system [23]. Microglia are mainly involved in neuroinflammatory reaction mediated by environmental or microbial stimuli. Activated microglia promote the production of neurotoxin(s), thus leading to neuronal cell death [24].

In our study, we explored the effects of JDS and its seven herbal components on neurodegeneration. First, the neuroprotective effects of herbal extracts of JDS and its seven components were determined using the HT22 hippocampal cell line. To induce neuronal cell damage, HT22 cells were exposed to H_2_O_2_ according to the accumulating evidence of H_2_O_2_-mediated neuronal cell death and alternation of redox signaling [25–27]. In the presence of concurrent treatment with H_2_O_2_ and each of the herbal extracts, JDS had a significant effect of inhibition of neuronal cell death in H_2_O_2_-treated hippocampal cells. Its components US, CM, ZO, PT, CU, and PC also displayed neuroprotective effects that were concentration dependent. US treatment significantly reversed cell viability at lower concentrations (90.91% ± 3.42% and 82.78% ± 4.97% at 12.5 and 25 *μ*g/mL, resp.) compared with H_2_O_2_-treated cells (53.00% ± 3.92%). However, cotreatment with H_2_O_2_ and a higher concentration of US significantly increased the level of H_2_O_2_-induced cell death. ZO significantly blocked neuronal cell death at 2.5 and 5 *μ*g/mL of the treatment (97.96% ± 13.57% and 84.74% ± 11.07%, resp.) compared with the H_2_O_2_ control (55.20% ± 2.48%). The effect of PC was not strong compared to that of the other extracts. In cells that were cotreated with H_2_O_2_ and PC at 25 *μ*g/mL, a 1.58-fold increase was observed compared to that observed for the treatment with H_2_O_2_ alone (76.83% ± 2.45% versus 48.53% ± 3.80%). CM, PT, and CU significantly protected against H_2_O_2_-induced cell death at a concentration ranging from 25 to 100 *μ*g/mL.

Microglia that are activated by neuronal injury stimulate the release of inflammatory cytokines, such as TNF-*α* and IL-6 [24, 28]. Thus, we assessed the production of TNF-*α* and IL-6 to examine the antineuroinflammatory activities of JDS and its herbal components. The inflammatory reaction was stimulated by adding LPS to microglia. As expected, LPS stimulation significantly enhanced the production of the cytokines TNF-*α* and IL-6. The herbal mixture JDS significantly reduced the LPS-stimulated increase in cytokine generation observed in BV-2 cells. Among the seven components, ZO, PG, PT, CU, and PC had inhibitory effects on the production of TNF-*α* and IL-6, and PC had the most dramatic effect of all the drugs tested.

Overall, our data demonstrate that JDS has neuroprotective and antineuroinflammatory effects. Among its seven components, ZO, PT, CU, and PC were commonly effective regarding both neuroprotection and anti-inflammation. CM and US were effective regarding neuroprotection, but not antineuroinflammation, whereas PG inhibited the inflammatory reaction with no neuroprotective activity. These data suggest that the combination of each component in the JDS herbal formula can accelerate the pharmacological activity of the drugs against neurodegeneration via a synergistic interaction.

Moreover, a simultaneous analysis of the standard compounds of JDS was performed to build up a quality control for JDS. JDS consists of seven medicinal herbs: US, ZO, CM, PG, PT, CU, and PC [12, 13]. The main chemical constituents of each herbal medicine are as follows: corynoxeine, hirsutine, ursolic acid, and physcion in US [29, 30], 6-gingerol and 6-shogaol in ZO [31], luteolin, apigenin, camphene, and parthenolide in CM [32, 33], ginsenoside Rg1 and ginsenoside Rb1 in PG [34], 3,4-dihydroxybenzaldehyde, methyleugenol, and 5′-*S*-5′-thioadenosine from PT [35], hesperidin, esculetin, nomilin, and narirutin in CU [36], and pachymic acid, eburicol, and eburicoic acid in PC [37]. Among those compounds, we analyzed 6-gingerol, luteolin, apigenin, hesperidin, and narirutin using HPLC-PDA. The established HPLC-PDA method was successfully applied for the simultaneous analysis of the five compounds of the JDS extract. As a result, hesperidin (37.892 ± 1.228 mg/g) was determined as being the most abundant compound of JDS.

In conclusion, the* in vitro* or* in vivo* efficacies of each standard compound of JDS on neurodegenerative diseases, including Alzheimer's disease, have been reported [38–41]. However, the combinatorial effect of the standard chemicals has never been considered. Further studies will be necessary to address the advantages of using herbal formulas compared to the administration of single herbs. In addition, the investigation of the efficacy of JDS should be expanded over various neurodegenerative diseases, as, to date, it has focused on dementia or the cognitive disorders of neurodegenerative diseases. The molecular mechanisms that underlie their effects should also be studied to confirm their potential as therapeutic agents for neurodegenerative diseases.

## Figures and Tables

**Table 1:** Herbal composition of JDS.

Latin name	Scientific name	Amount (g)	Origin
Uncariae Ramulus et Uncus	*Uncaria sinensis *(US)	9	China
Chrysanthemi Flos	*Chrysanthemum morifolium *(CM)	6	Namwon, Korea
Zingiberis Rhizoma	*Zingiber officinale *(ZO)	3	Andong, Korea
Ginseng Radix Alba	*Panax ginseng *(PG)	6	Punggi, Korea
Pinelliae Tuber	*Pinellia ternata *(PT)	9	China
Citri Unshii Pericarpium	*Citrus unshiu *(CU)	9	Jeju, Korea
Poria Sclerotium	*Poria cocos *(PC)	9	China
Total amount		51	

**Table 2:** Linear range, regression equation, correlation coefficients, LODs, and LOQs for compounds.

Compound	Linear range (*μ*g/mL)	Regression equation (*y* = *ax* + *b*)^(a)^		Correlation coefficient (*r*^2^)	LOD^(b)^ (*μ*g/mL)	LOQ^(c)^ (*μ*g/mL)
		Slope (*a*)	Intercept (*b*)			
Narirutin	12.5–400	18206	63887	0.9998	0.308	0.932
Hesperidin	31.25–500	20407	91283	0.9999	1.054	3.194
Luteolin	1.5625–25	40612	3770.9	1.0000	0.038	0.114
Apigenin	0.78125–25	81186	13562	0.9999	0.013	0.041
6-Gingerol	3.125–50	6274.2	2670.6	1.0000	0.175	0.531

^(a)^ *y* = *ax* + *b*, *y* means peak area and *x* means concentration (*μ*g/mL); ^(b)^LOD (Limit of detection): 3.3 × (SD of the response/slope of the calibration curve); ^(c)^LOQ (Limit of quantitation): 10 × (SD of the response/slope of the calibration curve).

**Table 3:** The content of standard compounds in JDS.

Compound	Content (mg/g)
Narirutin	12.289 ± 0.393
Hesperidin	37.892 ± 1.228
Luteolin	0.626 ± 0.026
Apigenin	0.061 ± 0.003
6-Gingerol	1.731 ± 0.038

**Table 4:** Cytotoxicity of JDS and its herbal components against HT22 and BV-2 cells (% of control)^(a)^.

Cells	*μ*g/mL	12.5		25		50		100	
		Mean	SEM^(b)^	Mean	SEM	Mean	SEM	Mean	SEM
HT22	JDS	102.20	1.77	103.15	3.38	100.16	4.42	91.96	5.34
	US	95.87	1.85	92.96	1.36	95.87	2.52	85.72	2.34
	CM	104.71	1.62	95.27	0.84	88.40	1.69	82.05	0.81
	ZO	97.89	1.82	91.13	0.94	92.21	4.85	77.90	3.35
	PG	103.40	1.20	105.66	1.90	103.74	2.34	100.03	2.30
	PT	105.27	1.92	110.40	1.29	114.69	1.57	125.25	1.60
	CU	96.42	1.29	97.49	0.96	100.73	1.53	109.72	2.90
	PC	93.86	0.80	94.23	1.84	91.99	1.68	84.93	1.44

BV-2	JDS	97.98	2.52	99.30	2.21	95.31	5.27	94.53	3.29
	US	108.86	4.90	107.62	3.14	109.28	7.00	88.21	3.84
	CM	94.64	6.92	89.48	2.78	77.09	4.26	75.22	2.66
	ZO	97.21	3.72	94.25	3.63	88.73	8.35	60.73	1.63
	PG	120.32	7.98	108.30	4.20	106.74	5.41	93.96	3.13
	PT	128.87	2.90	123.15	3.95	142.12	6.39	143.02	4.74
	CU	112.59	5.99	108.59	10.16	109.73	3.94	107.30	2.15
	PC	132.52	10.35	111.75	6.64	111.81	13.52	100.52	10.66

^(a)^The results are expressed as mean ± SEM of three independent experiments; ^(b)^SEM, standard error of the mean.

## References

[1] Przedborski S., Vila M., Jackson-Lewis V. (2003). Neurodegeneration: what is it and where are we?. *The Journal of Clinical Investigation*.

[2] Thompson L. M. (2008). Neurodegeneration: A question of balance. *Nature*.

[3] Deary I. J., Corley J., Gow A. J. (2009). Age-associated cognitive decline. *British Medical Bulletin*.

[4] Mae M., Armulik A., Betsholtz C. (2011). Getting to know the cast—cellular interactions and signaling at the neurovascular unit. *Current Pharmaceutical Design*.

[5] Stem M. S., Gardner T. W. (2013). Neurodegeneration in the pathogenesis of diabetic retinopathy: molecular mechanisms and therapeutic implications. *Current Medicinal Chemistry*.

[6] Chen Y., Li Q., Pan C. (2015). QiShenYiQi Pills, a compound in Chinese medicine, protects against pressure overload-induced cardiac hypertrophy through a multi-component and multi-target mode. *Scientific Reports*.

[7] Tian X., Liu L. (2012). Drug discovery enters a new era with multi-target intervention strategy. *Chinese Journal of Integrative Medicine*.

[8] Ablat N., Lv D., Ren R. (2016). Neuroprotective effects of a standardized flavonoid extract from safflower against a rotenone-induced rat model of Parkinson's disease. *Molecules*.

[9] Chang K.-H., Chen I.-C., Lin H.-Y. (2016). The aqueous extract of Glycyrrhiza inflata can upregulate unfolded protein response-mediated chaperones to reduce tau misfolding in cell models of Alzheimer’s disease. *Drug Design, Development and Therapy*.

[10] Li J., Jiang Z., Li X. (2015). Natural therapeutic agents for neurodegenerative diseases from a traditional herbal medicine Pongamia pinnata (L.) Pierre. *Bioorganic & Medicinal Chemistry Letters*.

[11] Shimada Y., Yang Q., Yokoyama K. (2003). Choto-san prevents occurence of stroke and prolongs life span in stroke-prone spontaneously hypertensive rats. *American Journal of Chinese Medicine*.

[12] Suzuki T., Futami S., Igari Y. (2005). A Chinese herbal medicine, choto-san, improves cognitive function and activities of daily living of patients with dementia: a double-blind, randomized, placebo-controlled study. *Journal of the American Geriatrics Society*.

[13] Watanabe H., Zhao Q., Matsumoto K. (2003). Pharmacological evidence for antidementia effect of Choto-san (Gouteng-san), a traditional Kampo medicine. *Pharmacology Biochemistry & Behavior*.

[14] Dohi K., Aruga T., Satoh K., Shioda S. (2004). Choto-san (Kampo Medicine) for the Treatment of Headache. *Headache: The Journal of Head and Face Pain*.

[15] Yamaguchi S., Matsubara M., Kobayashi S. (2004). Event-related brain potential changes after Choto-san administration in stroke patients with mild cognitive impairments. *Psychopharmacology*.

[16] Hayashi H., Tohda M., Watanabe H., Murakami Y., Matsumoto K. (2005). The effects of Choto-san on the mRNA expression of Alzheimer's disease related factors in the permanent ischemic rat brain. *Biological & Pharmaceutical Bulletin*.

[17] Sasaki-Hamada S., Tamaki K., Otsuka H. (2014). Chotosan, a kampo formula, ameliorates hippocampal LTD and cognitive deficits in juvenile-onset diabetes rats. *Journal of Pharmacological Sciences*.

[18] Wei M., Chen L., Liu J., Zhao J., Liu W., Feng F. (2016). Protective effects of a Chotosan fraction and its active components on *β*-amyloid-induced neurotoxicity. *Neuroscience Letters*.

[19] Lim H.-S., Jin S.-E., Kim O.-S., Shin H.-K., Jeong S.-J. (2015). Alantolactone from Saussurea lappa Exerts Antiinflammatory Effects by Inhibiting Chemokine Production and STAT1 Phosphorylation in TNF-*α* and IFN-*γ*-induced in HaCaT cells. *Phytotherapy Research*.

[20] Gorman A. M. (2008). Neuronal cell death in neurodegenerative diseases: recurring themes around protein handling: Apoptosis Review Series. *Journal of Cellular and Molecular Medicine*.

[21] DiMauro S., Schon E. A. (2008). Mitochondrial disorders in the nervous system. *Annual Review of Neuroscience*.

[22] Lin M. T., Beal M. F. (2006). Mitochondrial dysfunction and oxidative stress in neurodegenerative diseases. *Nature*.

[23] Gendelman H. E. (2002). Neural immunity: Friend or foe?. *Journal of NeuroVirology*.

[24] Chen W.-W., Zhang X., Huang W.-J. (2016). Role of neuroinflammation in neurodegenerative diseases (Review). *Molecular Medicine Reports*.

[25] Choi E.-O., Jeong J.-W., Park C. (2016). Baicalein protects C6 glial cells against hydrogen peroxide-induced oxidative stress and apoptosis through regulation of the Nrf2 signaling pathway. *International Journal of Molecular Medicine*.

[26] Lee H. J., Han J., Jang Y. (2015). Docosahexaenoic acid prevents paraquat-induced reactive oxygen species production in dopaminergic neurons via enhancement of glutathione homeostasis. *Biochemical and Biophysical Research Communications*.

[27] Song Q., Gou W.-L., Zhang R. (2015). FAM3A protects HT22 cells against hydrogen peroxide-induced oxidative stress through activation of PI3K/Akt but not MEK/ERK pathway. *Cellular Physiology and Biochemistry*.

[28] Rochfort K. D., Cummins P. M. (2015). The blood-brain barrier endothelium: A target for pro-inflammatory cytokines. *Biochemical Society Transactions*.

[29] Shimada Y., Goto H., Itoh T. (1999). Evaluation of the protective effects of alkaloids isolated from the hooks and stems of *Uncaria sinensis* on glutamate-induced neuronal death in cultured cerebellar granule cells from rats. *Journal of Pharmacy and Pharmacology*.

[30] Miyazawa M., Okuno Y., Imanishi K. (2005). Suppression of the SOS-inducing activity of mutagenic heterocyclic amine, Trp-P-1, by triterpenoid from Uncaria sinensis in the Salmonella typhimurium TA1535/pSK1002 umu test. *Journal of Agricultural and Food Chemistry*.

[31] Zick S. M., Ruffin M. T., Djuric Z., Normolle D., Brenner D. E. (2010). Quantitation of 6-, 8- and 10-gingerols and 6-shogaol in human plasma by high performance liquid chromatography with electrochemical detection. *International Journal of Biomedical Science*.

[32] Chen L., Kotani A., Kusu F., Wang Z., Zhu J., Hakamata H. (2015). Quantitative comparison of caffeoylquinic acids and flavonoids in Chrysanthemum morifolium flowers and their sulfur-fumigated products by three-channel liquid chromatography with electrochemical detection. *Chemical & Pharmaceutical Bulletin*.

[33] Qin S., Wen X. (2011). Simultaneous determination of 6 active components in Chrysanthemum morifolium by HPLC. *Zhongguo Zhongyao Zazhi*.

[34] Brown P. N., Yu R. (2013). Determination of ginsenoside content in panax ginseng C.A. meyer and panax quinquefolius L. root materials and finished products by high-performance liquid chromatography with ultraviolet absorbance detection: Interlaboratory study. *Journal of AOAC International*.

[35] Seo C.-S., Shin H.-K. (2016). HPLC-PDA method for simultaneous determination of nine marker components in banhasasim-tang. *Journal of Chromatographic Science (JCS)*.

[36] Kim H. G., Kim G.-S., Lee J. H. (2011). Determination of the change of flavonoid components as the defence materials of Citrus unshiu Marc. fruit peel against Penicillium digitatum by liquid chromatography coupled with tandem mass spectrometry. *Food Chemistry*.

[37] Xia B., Zhou Y., Tan H. S., Ding L. S., Xu H. X. (2014). Advanced ultra-performance liquid chromatography-photodiode array-quadrupole time-of-flight mass spectrometric methods for simultaneous screening and quantification of triterpenoids in Poria cocos. *Food Chemistry*.

[38] Park Y.-G., Park S.-Y. (2014). Gingerol prevents prion protein-mediated neuronal toxicity by regulating HIF prolyl hydroxylase 2 and prion protein. *International Journal of Molecular Medicine*.

[39] Wang H., Wang H., Cheng H., Che Z. (2016). Ameliorating effect of luteolin on memory impairment in an Alzheimer's disease model. *Molecular Medicine Reports*.

[40] Balez R., Steiner N., Engel M. (2016). Neuroprotective effects of apigenin against inflammation, neuronal excitability and apoptosis in an induced pluripotent stem cell model of Alzheimer’s disease. *Scientific Reports*.

[41] Justin Thenmozhi A., William Raja T. R., Manivasagam T., Janakiraman U., Essa M. M. (2016). Hesperidin ameliorates cognitive dysfunction, oxidative stress and apoptosis against aluminium chloride induced rat model of Alzheimer's disease. *Nutritional Neuroscience*.

